# Management of Poor Treatment Adherence in Teenage-Onset Bipolar I Disorder Using Long-Acting Injectable Second-Generation Antipsychotics: A Case Report

**DOI:** 10.7759/cureus.104186

**Published:** 2026-02-24

**Authors:** Swetha Raghavendra Prasad, Natarajan S

**Affiliations:** 1 Psychiatry, Sri Ramachandra Institute of Higher Education and Research, Chennai, IND

**Keywords:** bipolar disorder, case report, long-acting injectable antipsychotics, relapse prevention, treatment adherence

## Abstract

Bipolar disorder is a chronic episodic illness in which poor treatment adherence is a major contributor to relapse, repeated hospitalization, and functional decline. We report the case of a 22-year-old self-employed man, diagnosed with bipolar I disorder at 16 years of age, who presented with irritability, aggressive behavior, reduced need for sleep, and increased goal-directed activity. He had experienced multiple manic and depressive episodes over a six-year period, largely related to repeated discontinuation of oral psychotropic medications following symptomatic improvement, and had a positive family history of mood disorder. During the current episode, clinical stabilization was achieved with electroconvulsive therapy and oral pharmacotherapy, after which a long-acting injectable second-generation antipsychotic paliperidone was initiated at discharge, and a two-year follow-up was done with clinical visits till date to address persistent non-adherence. This case highlights the potential role of long-acting injectable antipsychotics in improving treatment continuity and reducing relapse risk in selected patients with bipolar I disorder and a history of poor adherence.

## Introduction

Bipolar disorder is a severe, chronic mental illness characterized by recurrent episodes of mania, hypomania, and depression, leading to substantial personal, social, and occupational impairment [1]. Long-term pharmacological maintenance is essential for sustained remission. However, poor adherence to treatment among patients is a major challenge in clinical practice.

Globally, bipolar disorder accounts for approximately 6.8% of disability-adjusted life years (DALYs) attributed to mental disorders [2]. Data from the Global Burden of Disease Study 2017 indicate a marked increase in the incidence and disease burden of bipolar disorder over recent decades, with DALYs rising by more than 50% between 1990 and 2017 [3]. Recurrent mood episodes are associated with neuroprogression, treatment resistance, and worsening functional outcomes, often conceptualized through the kindling hypothesis.

Second-generation long-acting injectable (LAI) antipsychotics have been extensively used in schizophrenia and are increasingly being explored in bipolar disorder, particularly for patients with poor insight and non-adherence. Despite emerging evidence, their use in bipolar disorder remains relatively underutilized in routine clinical practice [4,5]. From this perspective, we present a case that highlights the clinical utility of LAI antipsychotics in a poorly adherent patient with bipolar I disorder. International Classification of Diseases, 10th Revision (ICD-10) standards for diagnosis classification and the Young Mania Rating Scale (YMRS) for symptom assessment were used.

## Case presentation

A 22-year-old self-employed man was brought to the psychiatry department by his family with a 10-day history of irritability, aggressive behavior, decreased need for sleep, increased energy, and excessive traveling without informing family members. He had been verbally and physically abusive toward his parents and had damaged household property. The patient had no significant past medical history or comorbidities, including endocrine or neurological disorders, and denied the use of other drugs such as steroids. A family history of bipolar disorder was reported in a paternal uncle. ICD-10 F30.2, with symptoms of delusions noted in the patient, leads to the diagnosis of bipolar disorder. No significant history of drug abuse was reported. The urine drug test was positive for benzodiazepines, which were prescribed for him. Otherwise, there were no traces of drug usage. The kindling phenomenon without a stressor episode begets the episode. It is a form of neuroplasticity where repeated, initially subconvulsant stimuli to the brain (electrical or chemical) result in progressively more severe conditions. The significant trigger in the patient was a genetic contribution.

Ethical consent

Written and oral consent was obtained from both the patient and attender (his mother) for examination, case report publication, and presentation purposes.

Mental status examination and treatment

On examination, the patient was conscious, oriented, irritable, and overproductive, with increased psychomotor activity and impaired judgment. Vital parameters were within normal limits. The YMRS was followed for assessment, with a score of 26 at admission and 9 at discharge, which was helpful in assessing the symptoms. On follow-up, we used the Clinical Global Impression (CGI) score to assess functionality.

He was treated with six sessions of electroconvulsive therapy (ECT) in combination with oral risperidone 8 mg daily for one month. Additionally, injectable paliperidone was initiated with a 150 mg loading dose, followed by 100 mg on day seven, and subsequently maintained at 100 mg every 28 days. Through psychoeducation and follow-up, we observed a good clinical response. Given the history of repeated non-adherence, a LAI formulation of olanzapine was added as discharge medication. The treatment was adhered to continuously as physician care was available.

Psychoeducation

Parents’ misconceptions about psychiatric conditions and social stigma were addressed. The main concern is lifelong medication use and potential dependency. However, psychoeducation regarding the illness course and treatment adherence was provided to both the patient and his family. In our case, psychoeducation was the main factor that enlightened the patient and his family about the importance of adherence; hence, as clinicians, it is important to spend more time on psychoeducation, as it helps in significantly improving the outcome in a patient. The table displays each previous episode in the patient with the duration of adherence and relapse (Table 1). Detailed information regarding significant adverse effects during previous relapse episodes could not be obtained due to insufficient history documentation.

**Table 1:** Psychiatric history depicting presentation symptoms and treatment adherence

Year	Symptoms	Treatment	Adherence
2016, 16 years	Increased energy, risk-taking behavior, interpersonal conflicts, and reduced sleep. Lasted for six months, with non-adherence for three months	Oral mood stabilizers, antipsychotics, and four sessions of electroconvulsive therapy (ECT)	Discontinued in two months
2020, 20 years	Depressive episode following job loss during the COVID-19 pandemic, marked by low mood, social withdrawal, and reduced self-esteem. Lasted three months, with non-adherence for one month	Oral pharmacotherapy	Discontinued due to perceived recovery in eight months
2021, 21 years	Manic episode associated with excessive online gaming, financial difficulties, stalking his college mate to fall in love, and behavioral discontrol. Lasted three months, with non-adherence for one month	Five sessions of ECT along with oral psychotropic medications	Skipped doses, discontinued in eight months
2022, 22 years	Current manic episode with aggressive and disruptive behavior leading to hospitalization	Inpatient psychiatric management	Ongoing/under evaluation

## Discussion

Poor adherence to maintenance treatment remains one of the most significant determinants of relapse, hospitalization, and long-term disability in bipolar disorder patients. Non-adherence rates have been reported to range between 20% and 60%, often driven by poor insight, adverse effects, social stigma, and lack of perceived need for ongoing treatment. Recurrent mood episodes are associated with progressive functional decline, reduced quality of life, cognitive impairment, and increased caregiver burden, thereby reinforcing the chronic and relapsing nature of the illness [4].

In the present case, repeated discontinuation of oral pharmacotherapy led to frequent relapses necessitating multiple hospitalizations and ECT. This shows the clinical challenge posed due to treatment non-adherence, particularly in patients with early-onset illness and a highly recurrent course. Repeated mood episodes over a short duration have been associated with illness sensitization, treatment resistance, and poorer long-term outcomes, thus highlighting the importance of sustained mood stabilization [5].

LAI second-generation antipsychotics offer several advantages in this context, including assured medication delivery, reduced risk of relapse, improved treatment continuity, and enhanced clinician ability to monitor adherence. Emerging evidence suggests that LAIs may be beneficial in bipolar disorder patients with poor insight, frequent relapses, and a history of non-adherence, and may contribute to reduced hospitalization rates and improved functional outcomes when used early in the course of illness [4,5]. However, casual interference was not assessed in-depth in this study. The adverse effects of the drug include nausea, vomiting, prolactinoma, and extrapyramidal symptoms such as tardive dyskinesia. Hence, close clinician follow-up was maintained.

This case also emphasizes the importance of early identification of non-adherence patterns and proactive consideration of alternative treatment strategies. Rather than repeated cycles of relapse and crisis-driven interventions, timely initiation of long-acting drugs may help interrupt the relapse-rehospitalization cycle, thus reducing the illness burden [5]. The presence of genetic vulnerability, young age of onset, and multiple episodes within a short time frame further increased the risk of illness progression in this patient, supporting the need for an assertive and individualized treatment approach [4].

While ECT remains an effective option for acute severe episodes, particularly in treatment-resistant cases, it does not address the underlying issue of long-term adherence. Further longitudinal studies are needed to clarify the optimal timing, selection, and long-term outcomes of LAI use in bipolar disorder; however, this case adds to the growing clinical evidence supporting their role in carefully selected patients [5].

Recent studies have shown that the FDA has approved nine drugs for LAI use, which would improve clinical outcomes in such patients [6]

Limitations

This was a single case study, and a randomized clinical trial was not conducted; therefore, confounding factors could not be controlled and causal inference cannot be established.

## Conclusions

The patient and attender were advised to visit every 28 days to maintain the injectable drug administration. The years of follow-up are based on the number of episodes, the CGI score, and the compliance of the patient. In our case, we had a two-year follow-up, and the patient is still adherent mainly due to psychoeducation.

 Second-generation LAI antipsychotics may represent a valuable maintenance option for patients with bipolar I disorder who demonstrate persistent non-adherence to oral medications. Judicious use of LAIs, along with psychoeducation and family involvement, is expected to reduce relapse frequency and improve long-term outcomes among patients. However, this study is based on a hypothesis and an outcome obtained in one such non-adherent case; further studies and randomized controlled trials are needed to establish a significant conclusion on LAI's effect on non-adherent treatment.
